# Do managed alcohol programs change patterns of alcohol consumption and reduce related harm? A pilot study

**DOI:** 10.1186/s12954-016-0103-4

**Published:** 2016-05-09

**Authors:** Kate Vallance, Tim Stockwell, Bernie Pauly, Clifton Chow, Erin Gray, Bonnie Krysowaty, Kathleen Perkin, Jinhui Zhao

**Affiliations:** Centre for Addictions Research of BC, PO Box 1700 STN CSC, Victoria, BC V8W 2Y2 Canada; School of Social Work, MacEwan University, 10700-104 Avenue, Edmonton, AB T5J 2P2 Canada

**Keywords:** Alcohol harm reduction, Managed alcohol programs, Hospital admissions, Police contacts, Non-beverage alcohol, Non-potable alcohol, Housing First, Homelessness, Alcohol use disorders

## Abstract

**Background:**

Managed alcohol programs (MAPs) are a harm reduction strategy for people with severe alcohol dependence and unstable housing. MAPs provide controlled access to alcohol usually alongside accommodation, meals, and other supports. Patterns of alcohol consumption and related harms among MAP participants and controls from a homeless shelter in Thunder Bay, Ontario, were investigated in 2013.

**Methods:**

Structured interviews were conducted with 18 MAP and 20 control participants assessed as alcohol dependent with most using non-beverage alcohol (NBA). Qualitative interviews were conducted with seven participants and four MAP staff concerning perceptions and experiences of the program. Program alcohol consumption records were obtained for MAP participants, and records of police contacts and use of health services were obtained for participants and controls. Some participants’ liver function test (LFT) results were available for before and after MAP entry.

**Results:**

Compared with periods off the MAP, MAP participants had 41 % fewer police contacts, 33 % fewer police contacts leading to custody time (*x*^2^ = 43.84, *P* < 0.001), 87 % fewer detox admissions (*t* = −1.68, *P* = 0.06), and 32 % fewer hospital admissions (*t* = −2.08, *P* = 0.03). MAP and control participants shared similar characteristics, indicating the groups were broadly comparable. There were reductions in nearly all available LFT scores after MAP entry. Compared with controls, MAP participants had 43 % fewer police contacts, significantly fewer police contacts (−38 %) that resulted in custody time (*x*^2^ = 66.10, *P* < 0.001), 70 % fewer detox admissions (*t* = −2.19, *P* = 0.02), and 47 % fewer emergency room presentations. NBA use was significantly less frequent for MAP participants versus controls (*t* = −2.34, *P* < 0.05). Marked but non-significant reductions were observed in the number of participants self-reporting alcohol-related harms in the domains of home life, legal issues, and withdrawal seizures. Qualitative interviews with staff and MAP participants provided additional insight into reductions of non-beverage alcohol use and reductions of police and health-care contacts. It was unclear if overall volume of alcohol consumption was reduced as a result of MAP participation.

**Conclusions:**

The quantitative and qualitative findings of this pilot study suggest that MAP participation was associated with a number of positive outcomes including fewer hospital admissions, detox episodes, and police contacts leading to custody, reduced NBA consumption, and decreases in some alcohol-related harms. These encouraging trends are being investigated in a larger national study.

## Background

Severe alcohol dependence almost invariably carries heavy health and social costs and is sometimes associated with homelessness or housing instability [1–3]. In general, those who are severely dependent on alcohol and experiencing homelessness face significant barriers to accessing temporary accommodation and in some cases will go without shelter as a consequence of alcohol use [4].

There are many acute and chronic health, as well as social consequences associated with severe alcohol dependence, including increased risk of numerous physical diseases along with self-inflicted or accidental injuries and experiences of violence [5, 6]. In some cases, non-beverage alcohol such as rubbing alcohol, mouthwash, or alcohol-based hand sanitizers may be consumed. These sources of alcohol are relatively low-cost and readily available and may contribute to a variety of additional health risks when consumed in large volumes [7–9]. There is only limited evidence that ingredients added to non-beverage alcohols pose risks to health over and above the significant risks associated with beverage alcohol consumption [10].

Methanol is known to be particularly hazardous while isopropanol, which is an ingredient found in rubbing alcohol and hand sanitizer, also poses potential risks but to a lesser degree [11, 12]. Concentration levels of ethanol in certain types of non-beverage alcohol (NBA) are extremely high, and, for example, consumption of one 500 ml bottle of 95 % rubbing alcohol is the equivalent of about 28 standard drinks. Dental mouthwashes such as Listerine (which contain 26.9 % alcohol/volume) are not thought to pose serious risks unless consumed at very high doses; however, this is more likely to occur among more unstably housed and severely alcohol-dependent populations [9]. There is also a stigma associated with non-beverage alcohol use which can be damaging to self-esteem and overall mental health and can also decrease the likelihood of seeking treatment from health care and other mainstream treatment providers such as Alcoholics Anonymous [13].

There are few established programs for people experiencing both severe alcohol dependence and housing instability [14–21]. However, “Housing First” programs, which are a relatively recent innovation to address homelessness, seek to incorporate a harm reduction philosophy and practices to reduce the harms of substance use without necessarily eliminating or reducing use [16, 22–25]. Some programs seek to reduce harms for a particular population, primarily by providing stable housing, which can have intrinsic health and social benefits, and tolerating continued use of alcohol. Managed alcohol programs (MAPs) take this approach a step further by providing beverage alcohol of known quality to program participants at regular intervals to stabilize drinking patterns and to replace non-beverage alcohol which can be more hazardous.

Among the few published evaluations of programs that tolerate alcohol use, but do not provide or manage its availability, Thornquist et al. [21] reported that attending such programs resulted in decreased use of hospital and detoxification services. Other studies also reported that non-abstinence-based housing was associated with reductions in alcohol-related harm [15, 17, 26], as well as reductions in use of publicly funded services (e.g., hospital visits, jail, use of detox facilities, and emergency medical services) [17, 18, 27]. In Ottawa, an evaluation of 17 adults involved in the Ottawa MAP showed improved health outcomes, fewer emergency room (ER) visits, fewer police contacts, and reduced alcohol consumption over an average of 16 months in the program [19]. It is noteworthy, however, that there was no attempt to record participants’ alcohol consumption outside of the MAP in this study. Therefore, it is not clear whether overall consumption was in fact reduced.

A MAP was established in Thunder Bay, Ontario, in 2012, with the objective of reducing harms from alcohol use and improving overall quality of life for unstably housed individuals experiencing repeated and severe alcohol-related problems. Reduction of alcohol consumption was not identified as a main goal of the program, although a switch from use of NBA to beverage alcohol was encouraged. A small pilot evaluation was conducted in order to establish whether entry into the MAP was associated with (i) significant improvements in health and well-being, (ii) reductions in harms as indicated by decreased use of emergency, hospital, and police services, and (iii) less hazardous patterns of alcohol use as indicated by reduced use of non-beverage alcohol, fewer episodes of severe intoxication, and decreased consumption in high-risk drinking settings without an overall increase in alcohol consumption. In this paper, we present analyses of changes in alcohol use, related harms and use of police, and health-care services for the MAP participants and for a group of control participants assessed as meeting the entry criteria for the MAP. A companion paper [28] explores associated changes in housing stability, housing satisfaction, and quality of life.

### Description of program

Shelter House’s Kwae Kii Win Centre in Thunder Bay, Ontario, is a 15-bed mixed-gender MAP that was established in March 2012 in response to the needs of people in the community with severe and chronic alcohol use problems, many of whom have long histories of homelessness, public intoxication, and regularly consume non-beverage alcohol. The program was created in an effort to reduce alcohol-related harm for program participants and to alleviate the load on police and emergency responders in dealing with public intoxication. The intention of the program is to replace a dangerous pattern of episodic, very heavy drinking, and non-beverage alcohol drinking, with maintenance doses of beverage alcohol in a supervised setting. Residents receive stable housing, meals, help managing money, access to primary health care, life skills training, counseling, and one alcoholic drink every 90 min from 8 am to 11 pm. The program generally uses 12 % alcohol/volume white wine, and each drink served is 6 oz, i.e., 20.46 ml or 16.14 g of ethanol. To receive each dose, participants must not be overly intoxicated and must have been present at the facility for at least 60 min prior. Drinking outside the program is discouraged and participants are not allowed to store their alcohol on-site for later consumption.

Individuals participating in the MAP are provided housing, similar in style to that of a rooming house with communal living spaces. Residential tenure is contingent on their participation in the MAP. Participants are provided meals and access to food and groceries whenever they choose and are able to remain indoors or sleep during the day. Participants are offered recreational activities on-site, and transportation to activities in the community is provided. Residents in the MAP are also encouraged to see health-care workers such as nurses, doctors, and psychiatrists on a regular basis, and transportation is provided for off-site appointments. An Elder visits the program weekly to speak with MAP participants, other Indigenous-focused activities such as drumming are provided through the shelter program next door, and MAP residents are encouraged to attend. Participants sometimes leave seasonally for specific periods of time to visit family in more remote areas of the region and in some cases may be required to leave the program due to contravention of program rules or due to incarceration or hospital stays. In the case of hospital stays, a participant’s spot is held for them until such time as they can return to the program. Criteria for admission to the program include severe alcohol dependence, chronic homelessness, and a high rate of police contacts. When a spot in the MAP becomes available, staff from the shelter next door or the police may identify an individual for consideration who also fits the admission criteria and who is at high risk of injury, illness, or death if they remain on the street. However, there was a very low turnover of people on the MAP [28]. All program participants at the time of the pilot study identified as Indigenous.

## Methods

### Sampling and recruitment

We conducted this small scale study using a mixed methods research design with a non-randomized controlled sampling strategy as a pilot for a larger national evaluation of MAPs in Canada. Quantitative data on outcomes of interest were collected from male and female participants in a series of short monthly surveys, with more in-depth surveys at baseline and 6-month follow-ups. Qualitative interviews were conducted with MAP staff and residents to gain a deeper understanding of their experiences within the program. A mixed methods design incorporates multiple methods of inquiry to enhance scope and breadth of understanding in evaluation research [29, 30]. Both triangulation (convergence of findings) and complementarity (examining different aspects of the same phenomena) were employed within this study. A mixed-gender control group was included to help determine whether observed benefits or harms experienced by participants were specifically due to participation in the MAP. Control participants were recruited by the study researchers at the shelter next door to the MAP, which was a separate facility from the MAP but run by the same agency. The shelter provides access to set meals three times a day, and weather permitting, participants must be outside the building during the day other than at staff discretion if there is a risk of harm from being outside. There are no organized recreational activities offered at the shelter, and rides may be available for health-care appointments after 2 pm each day, but access to transportation is not guaranteed. Some Indigenous-focused activities such as drumming circles are also available, but there are no regular visits by Elders.

In order to be eligible to participate in the study, control participants had to meet the criteria for potential entry into the MAP but due to program space or personal choice, not currently be participants in the MAP. The proportion of controls that had not entered the MAP due to program space versus personal choice is not known. Participant reasons for not entering MAP included perceptions of stigma related to being administered alcohol and also some potential discomfort with having to follow rules within a more structured program setting. It was not feasible to randomly assign eligible individuals to the MAP or shelter controls because the MAP operates independently of the research study. The shelter and the MAP are run by the same organization and provide services to the same population. However, because the MAP was restricted to 15 beds and there was a low program turnover, many of the individuals attending the adjacent shelter would have been eligible for MAP and were therefore similar to a waiting-list control group. In order to locate comparable controls, the program staff initially compiled a list of individuals from the shelter who fit the criteria and would be eligible for the MAP but were not currently in the program. The first 15 controls who met the criteria were selected. The screening tool used to establish eligibility of controls consisted of four questions: (1) whether they had been without their own place to stay in the past 6 months, (2) whether they had been without their own place to stay more than four times in the past 6 months, (3) whether they have had many difficulties caused by drinking in the past few years, and (4) whether they had been picked up by the police due to alcohol, been to the hospital ER due to alcohol, or been to detox at least four times in total in the past 6 months. If they met each criterion, they were included in the study.

Quantitative data were collected for a 6-month period between March and September 2013. At the beginning of the 6-month period, structured quantitative baseline surveys were conducted with 18 consenting MAP and 20 consenting control participants in March and April of 2013. Fifteen of the 18 MAP participants had been residents in the program for at least 1 month when the first survey was conducted. Between the second and fifth month of data collection, a subset of six of the newly admitted MAP participants and 10 matched control participants were selected to complete shorter monthly quantitative follow-up interviews. Control participants were selected at a ratio of two MAP participants to three control participants to account for higher anticipated attrition among controls. At the 6-month mark, an in-depth quantitative survey was conducted with as many as possible of the smaller subset of MAP and control participants who had been selected for follow-up. Monthly follow-up surveys were conducted not only in part to gather information but also as a strategy to maintain contact with this population, especially the control participants who were more likely than MAP participants to be moving from place to place because of homelessness. One-time, face-to-face qualitative surveys were conducted with four MAP staff and seven MAP residents within the 6-month period in 2013.

Participation in the study was voluntary and written informed consent was obtained from all participants prior to their taking part. MAP and control participants received $25 gift vouchers for the longer quantitative interviews and $10 gift vouchers for the shorter, monthly quantitative follow-up interviews. MAP participants who completed the qualitative interviews received a $25 gift voucher. Ethical approval for this study was obtained from the University of Victoria and Lakehead University Human Research Ethics Committees, Thunder Bay Regional Health Science Centre (TBRHSC), and St. Joseph’s Care Group (SJCG) research ethics committees.

### Measures

#### Quantitative surveys

The quantitative surveys conducted at the beginning and end of the 6-month data collection period covered the following domains: sociodemographic characteristics; housing status over the past 12 months; alcohol and other substance use; severity of alcohol-related problems and degree of alcohol dependence; health and mental health; and housing quality. A range of questions on individual-level alcohol-related social harms was also included. Several standardized instruments were included in the survey such as the Alcohol Use Disorders Test (AUDIT) [31], the Severity of Alcohol Dependence Questionnaire (SADQ) [32], the Colorado Symptom Index [33] which measures psychological symptomatology, and the WHOQOL-BREF [34], which is an assessment of quality of life. The shorter quantitative surveys conducted monthly included questions about housing and alcohol consumption and also the WHOQOL-BREF. Housing quality and satisfaction as well as quality of life data are reported separately [28].

Results from the quantitative surveys presented in this paper primarily include data from the initial interviews conducted at the beginning of the study. As just six MAP participants and seven controls were successfully followed up and completed the longer interview at the 6-month mark, only limited descriptive data will be provided on their outcomes. Analysis of the survey data from the larger number of initial intake interviews included chi-square tests [35] to determine significant differences between the MAP participants and control participants on selected demographic variables, self-reported alcohol consumption, and a selection of relevant individual-level alcohol-related social harms such as home life, housing status, legal issues, and experience of withdrawal seizures. A two-sample *t* test was used to test any significant differences in reported AUDIT scores and days of NBA use.

#### MAP alcohol consumption records

Alcohol administration data routinely collected by MAP staff were accessed for the study, and these included the number of drinks administered per serve as well as the time of day that the serve occurred. A question was also included about consumption outside the MAP in the previous 24 hours and was asked of residents as an open-ended question at the time of their first drink of the day.

#### Liver function tests

Blood samples for liver functions tests (LFTs) were collected by a nurse practitioner from a nearby health clinic at intervals throughout the program. In some cases, LFT results from MAP and control participants’ health records were also available. LFT results accessed included aspartate transaminase (AST), a liver enzyme sensitive to acute liver damage with a normal range between five and 40; alanine transaminase (ALT), a liver enzyme with a normal range between seven and 56; and gamma glutamyl transpeptidase (GGT), which measures liver dysfunction and has a normal range of zero to 65 for males and zero to 45 in females. Factors other than alcohol consumption can affect liver functioning on these tests, e.g., hepatitis, nutrition, and body weight.

#### Police and health-care records

Ethical approval and written consent from 13 MAP participants and 10 controls was obtained to access archival police and health-care records for the 5 years prior to the initiation of the study and 12 months afterwards for both MAP and control participants. The date range for these records was August 2008 to August 2013. Police records from the Thunder Bay Police included the number of police contacts and in cases when police contacts resulted in custody or jail time, the length of custody and jail time. Individual level health-care records from the Thunder Bay Regional Health Science Centre included information on frequency and duration of hospital visits, and data from St. Joseph’s Care Group included in-patient detoxification episodes.

MAP participants averaged 357.5 days (SD = 321.47, Min/Max 398/1728) on the program compared to 1220.9 days (SD = 143.98, Min/Max 91/529) off the program during that 5-year time period. The numbers of police contacts, hospital admissions, ER presentations, and detoxifications per 100 observed days were estimated for participants on MAP and off MAP and for controls. We used one-sided paired *t* tests and two-sample *t* tests to test the hypotheses that MAP participation was associated with improved outcomes, compared with periods prior to MAP entry and compared with similar controls who were not on a MAP [35]. Paired *t* tests were used to investigate any significant difference in the numbers of police contacts, hospital admissions, ER presentations, and detoxifications per 100 observed days between participants on MAP and off MAP. Two-sample *t* tests were used to investigate observed differences in rates of police contacts, hospital admissions, ER presentations, and detoxifications per 100 observed days between participants on MAP and controls. In each case, one-sided significance tests were employed to test explicit hypotheses that MAP participation would be associated with reductions in these areas. Chi-square (*x*^2^) tests were used to compare the proportions of police contacts leading to custody time for participants while on the MAP compared with periods off the MAP as well as compared with controls.

#### Qualitative interviews

Experienced qualitative researchers from the study team conducted one-time face-to-face interviews with seven MAP residents, three females, and four males, all of whom identified as Indigenous, and who had been in the program at least 1 month. For MAP residents, the focus of the qualitative interview was on their experiences before entering the program, their experiences within the program, and the impact of the program on patterns of drinking, health, housing, quality of life, and social relationships. Interviews were conducted with four of the MAP staff with a focus on their experiences working in the program, including their thoughts on program goals and structures, changes in the program, program impacts, and community responses to the program. Qualitative findings related to health, quality of life, and housing are reported in the sister manuscript in this issue [28]. All interviews were audio-taped and transcribed. Qualitative interviews were not conducted with control participants.

Analysis of the qualitative data was conducted using constant comparative analysis, in which each transcript was read and re-read by two members of the research team and coded inductively by both research team members for key ideas and themes that described the experience of being in a MAP related to health, housing, quality of life, and harms of alcohol use and drinking patterns. An inductive coding framework was developed, and NVivo (NVivo qualitative data analysis software; QSR International Pty Ltd. Version 10, 2012) was used to organize and manage the data [36–39]. Qualitative results presented in this paper relate specifically to life in the MAP, use of NBA and alcohol consumption patterns, contacts with police, and access to and use of health-care services.

## Results

### Survey data: participant characteristics

#### MAP participants

Based on self-report data from baseline quantitative surveys, just over one third of MAP participants were female and all 18 self-identified as Aboriginal. Mean age was 42 years (range 25–61) and approximately one third of all participants had completed high school, over half had been married, and all reported being currently unemployed. Each of the MAP participants had been accessing a homeless shelter or living on the street prior to entering the MAP (see Table 1). Most of them reported AUDIT scores indicative of alcohol dependence with scores of 20 or greater (mean 28.5, range 17–36) [31].

**Table 1:** MAP and control participant characteristics

Characteristics	MAP (*n* = 18)	Controls (*n* = 20)
Age (mean, range)	41.7 (25–61)	36.8 (21–50)
Female (*n*, %)	7 (38.9 %)	8 (40 %)
Indigenous (*n*, %)	18 (100 %)	20 (100 %)
Finished high school	6 (33.3 %)	8 (40 %)
Married (*n*, %)	9 (55.6 %)	10 (50 %)
Unemployed (*n*, %)	18 (100 %)	20 (100 %)

Chi-square and paired *t* tests indicated no significant differences between MAP and control participants on these characteristics

#### Control participants

Eight or 40 % of control participants were female, and the rest identified as male. All 20 self-identified as Indigenous and mean age was 37 years (range 21–50). Forty percent of control participants reported completing high school, half had been married, and all were currently unemployed. Nearly all control participants reported staying in an emergency shelter the previous night. Most of them had AUDIT scores indicative of alcohol dependence, i.e., scores of 20 or greater (mean 31, range 16–40) [31].

*Chi-square* tests run on the demographic variables listed in Table 1 showed no significant differences between the MAP and control participants indicating that the two groups were broadly similar. In addition, mean values of the AUDIT scores for MAP participants and controls were also very similar (28.5 vs 31) indicating that overall the controls were an appropriate comparison group.

### Self-reported total alcohol consumption

MAP participants reported consuming alcohol on a significantly higher number of days out of the past month than control participants at 27.8 days versus 22.6 days (*t* test *P* > 0.05), likely reflecting the more consistent access to alcohol available to those on the MAP. MAP and control participants reported consuming a similar average number of drinks per day on days that they drank in the past month. Controls reported a slightly higher number (20.9 drinks/day) compared to MAP participants (19.1 drinks per day) (*t* test *P* > 0.05). These self-reports included MAP participants’ estimates of alcohol consumption on and off the MAP, including both beverage and non-beverage alcohol consumption. A high frequency of drinking (mean of 28/30 days) was maintained at 6 months among the five MAP participants with available data selected for follow-up, markedly higher than the average of 16/30 days for the six controls with available data followed up.

### Self-reported non-beverage alcohol use

The three most common types of non-beverage alcohol (NBA) consumed by both MAP residents and the controls were mouthwash, hand sanitizer, and hairspray. Other types of NBA reported being consumed included rubbing alcohol and cooking wine. Slightly more controls than MAP participants reported consuming NBA at least once in the previous month, although the difference was not significant. However, reports of NBA consumption in the past month showed that MAP participants consumed NBA on significantly fewer days (M = 4.3, SD = 5.9) than control participants (M = 12.4, SD = 13.8, (*t* test = - 2.34, *P* < 0.05). The decrease in frequency of NBA use by MAP participants was also noted in the qualitative interviews, and participants expressed a preference and desire to “avoid” NBA. Some participants made a strong distinction between the wine provided on the program and the “garbage” and “bad stuff” they had consumed prior to starting the program, indicating that their preference was to consume beverage alcohol when available.

As one MAP participant said,

I haven’t even drank that other, the hairspray in such a long time, I don’t even remember when, I think about seven months or something like that. And the other stuff, I refuse it now. You know I could still be drunk everyday if I wanted to, with the people I was on the street with. [But] since coming here I drink wine… I don’t think about buying anything else, you know like the other garbage like antiseptic, hairspray, all the other stuff that you know that I used to consume when I was out there.

MAP participants noted that they still had opportunities to access NBA but that having the opportunity to drink the wine provided by the program supported their efforts avoiding NBA and also helped them avoid feeling sick while still controlling their cravings for alcohol.

### Survey data: self-reported alcohol-related harms

**Fig. 1:**
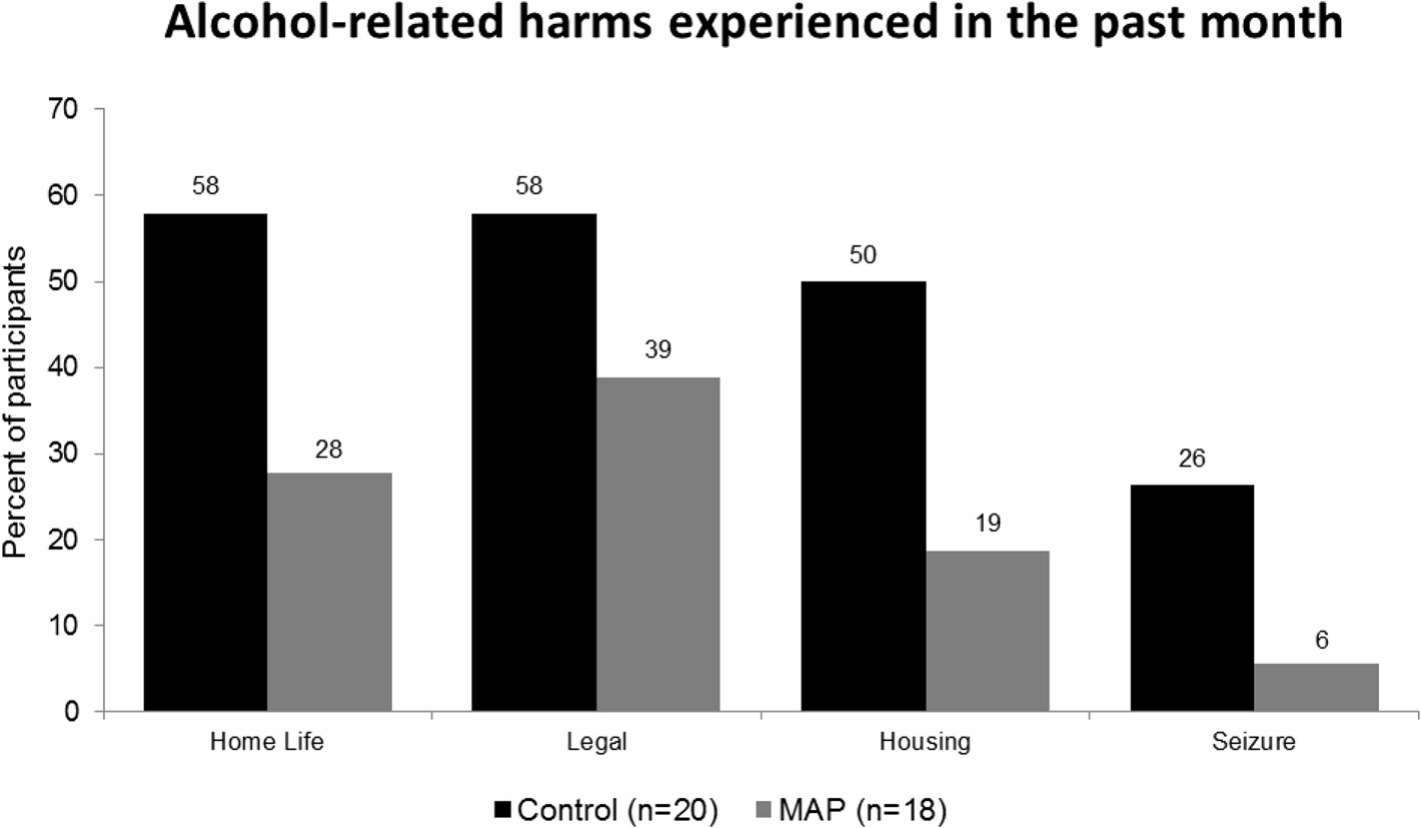
Alcohol-related harms experienced in the past month. Figure represents percentages of MAP participants and controls who experienced harms. Sample size was 20 control participants and 18 managed alcohol program (MAP) participants. There were no missing values

Fewer MAP participants reported alcohol-related harms in the last 30 days than controls in the domains of home life, legal issues, housing status, and experience of withdrawal seizures (see Fig. 1), though differences were not significant. In the qualitative interviews, several participants indicated strong feelings of increased safety in the MAP compared to their experience of harms on the street and in hospitals and jails. These findings are also reported in greater depth in the sister manuscript in this issue [28].

### MAP alcohol consumption records

Over the course of the 6 months of data collection, MAP participants were administered an average of 7.3 standard drinks per day by the program staff with little variation from month to month. Reports provided to staff of alcohol consumption outside the program over the 6-month period remained fairly steady with an average of 2.9 drinks per day. The average total number of drinks per day, including both drinks provided by the program and those consumed outside the program, was 10.4 drinks for MAP participants (see Table 2). It should be noted that the data on non-MAP drinks only apply to days that the participants attended the program lounge to be administered drinks by staff and who answered the questions about their alcohol consumption outside of the program on the previous day. Notably, the estimates of average total alcohol consumption gathered by staff in the alcohol administration data (10.4 drinks) are only about half of the total of MAP plus outside-MAP consumption reported by participants during their baseline interviews (19.1 drinks), which likely indicates an underestimation of outside-MAP drinks being reported to staff.

**Table 2:** Alcohol consumption records for MAP drinks and outside-MAP drinks, March–Sept 2013

	March	April	May	June	July	Aug	Sept	Total
MAP drinks	7.23	8.11	7.94	6.47	6.93	6.65	7.74	7.3
Non-MAP drinks	2.98	2.81	2.58	2.37	^a^	^a^	3.59	2.87
Avg. total drinks	10.21	10.93	10.52	8.84	^a^	^a^	11.37	10.37

^a^Records for outside-MAP drinks for July and August were incomplete and therefore excluded

### Liver function test results

Some liver function test results were available from previous health records for 13 MAP participants. Among those who had comparable repeated tests, seven of seven showed reductions in AST or remained within normal ranges (mean pre = 208, post = 84), nine out of ten had reductions in ALT or remained normal (mean pre = 72.5, mean post = 53.6), and one reduced GGT (1253 vs 803).

### Police and health-care records

#### MAP participants on versus off MAP

Table 3 presents the estimate of mean numbers of police contacts, hospital admissions, ER presentations and detox admissions per 100 observed days while on and off the MAP for 13 of the MAP participants between 2008 and 2013. Rates of mean detoxification (*t* test *P* = 0.06) and hospital admissions (*t* test *P* < 0.05) were lower when clients were on the MAP compared to off the MAP. Rates of police contacts were also lower when clients were on versus off the MAP, but the difference was not significant (*t* test *P* > 0.05). However, while participants were on the MAP, only 41.6 % of their police contacts resulted in custody time whereas while they were off the MAP, 74.4 % of their police contacts resulted in custody time, which was a significant difference (*x*^2^ = 43.84, *P* < 0.001).

**Table 3:** Mean numbers per 100 observed days, 2008–2013 for participants on and off MAP

Outcomes	Number	Participants on MAP	Participants off MAP	Change	Paired *t* test (one-tailed)	
		Mean (95 % CI)	Mean (95 % CI)	%	*t* statistic	*P* value
Police contact	13	2.79 (1.38–4.20)	4.77 (0.04–9.50)	−41.5	−0.90	0.194
Detoxification	13	0.33 (0.04–0.62)	2.46 (0.00–5.51)	−86.6	−1.68	0.060
Hospital admission	13	0.26 (0.14–0.38)	0.38 (0.00–0.79)	−31.6	−2.08	0.030
ER presentation	13	3.82 (2.10–5.54)	3.60 (1.65–5.55)	6	+0.22	0.586

#### MAP participants versus controls

Table 4 presents the estimates of mean numbers of police contacts, hospital and ER presentations, and detox admissions per 100 observed days among participants while they were on the MAP and among controls. The estimate of mean detoxification episodes was significantly lower among MAP participants than for controls (*P* = 0.02). The estimates of mean police contacts, hospital admissions, and ER presentations were markedly lower among MAP participants than among controls (see percent change in Table 4) but not statistically significantly so (*t* test *P* > 0.05). The proportion of police contacts for MAP participants that resulted in custody time was significantly lower when compared to controls (*x*^2^ = 66.10, *P* < 0.001). As mentioned above, only 41.6 % of police contacts while participants were on the MAP resulted in custody time, while 79.5 % of police contacts for control participants resulted in custody time.

**Table 4:** Mean numbers per 100 days, 2008–2013 for MAP participants and controls

Outcomes	Participants on MAP		Controls		Change	Two sample *t* test (one-sided)	
	*N*	Mean (95 % CI)	*N*	Mean (95 % CI)	%	*t* statistic	*P* value
Police contact	13	2.79 (1.38–4.20)	10	4.87 (0.00–10.31)	−42.7	−0.93	0.1804
Detoxification	13	0.33 (0.04–0.62)	10	1.59 (0.14–3.05)	−79.2	−2.19	0.0201
Hospital admission	13	0.26 (0.14–0.38)	10	0.42 (0.01–0.84)	−38.1	−0.97	0.1717
ER presentation	13	3.82 (2.10–5.54)	10	7.15 (0.00–17.71)	−46.6	−0.80	0.2165

#### Police contacts: qualitative data

In the qualitative interviews, MAP participants and staff spoke about the MAP as a better alternative than their previous repetitive negative experiences of police interactions or being taken to jail. Even more encouraging was that participation in the MAP had altered participants’ circumstances such that they no longer needed to partake in potentially harmful activities such as sleeping in abandoned vehicles and stealing non-beverage alcohol like mouthwash. One participant observed, “I used to go steal that mouthwash just to try and feel – get myself to feel better. I used to do that. I was in and out of jail. Ever since I’ve moved here, I haven’t even had any police contact.” Staff also noted that police, regardless of their personal perceptions of the program, would bring intoxicated residents back to the MAP instead of taking them to jail. Both MAP participants and staff described the MAP as a means of reducing negative police contacts and as a safer alternative for managing public intoxication compared to street-life, jails, or through police enforcement [28].

#### Health-care contacts: qualitative data

There were also indications from staff and MAP participants in the qualitative data that those in the MAP had both improved and more regular access to primary health-care services. There seemed to be a shift in focus from managing multiple health crises to addressing health issues on an ongoing basis. A key reason for the shift was attributed to the presence of a nurse practitioner who provided on-site health-care services to MAP participants. As the service was provided within the context and location of the program, MAP participants were less likely to access health care at the ER and were more able to manage previously unaddressed health issues and concerns. Many of the participants indicated that they had health issues that, prior to admission to the MAP, were not being addressed because they were not able to keep appointments or did not want to go to the hospital due to previous negative experiences. One participant talked about finally being able to have a surgical operation that had been repeatedly put off when he was homeless. MAP participants also expressed that staff members were helpful in assisting them with tracking appointments and providing necessary transportation when medical care was to be provided off-site. Although further analysis is needed, these data suggest that while ER and detox use were lower, use of more appropriate primary care services may have increased as clients were being facilitated to address previously neglected health issues.

## Discussion

In this paper, we present findings from a pilot evaluation of a managed alcohol program (MAP) in Thunder Bay, Ontario, to explore how participation in the MAP may have impacted a variety of alcohol-related patterns and harms for these participants who were previously homeless and experiencing severe alcohol dependency as compared to a similar population who were recruited as controls. A number of indicators suggested positive changes to alcohol consumption patterns and a reduction in a variety of alcohol-related harms while on the MAP, although small sample numbers limited conclusive statistical comparisons on some outcomes. Positive and significant outcomes for MAP participants included fewer days of non-beverage alcohol (NBA) use compared to controls (4.3 vs 12.4 days), fewer detoxification episodes compared to controls (a decrease of 79 %), fewer police contacts leading to custody time for participants on the MAP compared to off the MAP (a difference of −33 %) and compared to controls (a difference of -38 %), and fewer hospital admissions compared to when off MAP (a decrease of 32 %). MAP participation was also associated with sizeable though non-significant reductions across other dimensions including overall police contacts compared to when participants were off the MAP (a decrease of 42 %), hospital admission for MAP participants as compared to controls (a decrease of 38 %), and ER presentations for MAP participants as compared to controls (a decrease of 47 %).

Reports from the qualitative interviews of improved relationships with police and health-care providers as well as positive experiences of making the switch to beverage alcohol highlight the importance of MAP programs in altering the environment that contributes to alcohol-related harms. While participants have many more supports available to them as part of the MAP program than they would at the nearby shelter, it is likely that the consistency of the alcohol provided by MAP and the stabilization of their patterns of consumption facilitated them to be in a position to accept these supports with implications for positive changes. These reports were consistent with the interpretation that MAP participation is associated with reduced alcohol-related harm. This interpretation is further supported by the available data on liver function tests which showed almost uniformly reducing scores for available results pre-and post-entry into the MAP. Qualitative findings regarding the impact of the program on healing and recovery particularly for Indigenous participants are delved into more fully in the sister manuscript [28].

There is suggestive evidence from these limited pilot data that the context and pattern of drinking for many MAP participants may have been lower risk than for their peers on the street, though questions remain as to whether alcohol consumption overall was reduced for participants. The fact that residents were mostly consuming alcohol within the program as opposed to on the street and that the program drinks were spaced out through the day as opposed to consumed quickly all at once is indicative both of much safer patterns and contexts for drinking. The alcohol content of the wine available on the MAP was also much lower on average than what would normally be found in most types of NBA. However, the amount of alcohol consumed outside of the program was likely under-estimated, perhaps due to participants feeling uncomfortable reporting their drinking outside of the MAP to staff for fear of losing their place in the MAP. While it is clear that the pattern, context, and type of alcohol consumed were mostly less hazardous for the MAP participants, it is not possible to be certain that there was no increase in overall alcohol consumption as a result of their participation. Podymow et al.’s [19] evaluation of the Ottawa MAP indicated a gradual reduction in volume of alcohol consumption for participants. However, they only recorded alcohol provided by the MAP itself and did not investigate outside consumption. Furthermore, we observed a markedly higher frequency of drinking among MAP participants than controls among the small sample followed up, a trend to be investigated in the larger study but which likely reflects the ready availability of alcohol on the MAP program.

While consumption of NBA did continue, it was at a lower level for MAP participants than before entry into the program and also at lower levels compared to the control participants. The majority of the type of alcohol being consumed by the MAP participants was intrinsically less hazardous compared to the control sample, i.e., the wine provided on the program as opposed to a variety of forms of non-beverage and higher strength alcohol. The qualitative data also highlighted that the gradual shift from consumption of non-beverage alcohol to the types provided on the MAP clearly had an impact on participants’ concept of self as well as their overall health and well-being. Participant reports of making the switch to beverage alcohol, and reducing NBA consumption was seen as a positive step and often a source of pride. In addition to the smoothing out of the pattern of alcohol consumption due to the regular administration by the program, this change from non-beverage to beverage alcohol suggests that the MAP may have specific alcohol-related harm reduction benefits over and above what normally is found in Housing First initiatives where no administration of alcohol occurs.

Fewer MAP participants self-reported alcohol-related harms in four domains (legal issues, home life, withdrawal seizures, and housing status) compared with controls, and while these differences were not significant, they were substantiated by reports of improved quality of life from both MAP participants as well as observations from MAP staff. The analysis of police and health-care data obtained from the local police department and regional hospital indicated that the average number of police contacts and overall hospital-based health-care interactions (including hospital admissions, ER presentations, and detoxification episodes) were lower for MAP participants when they were on the program as opposed to off the program, although only the reduction in hospital admissions proved statistically significant. The average number of police contacts and hospital-based health-care interactions was also lower for MAP participants compared to the control participants, although again not significantly so. However, the MAP participants had significantly fewer average detoxification episodes than the control participants. The MAP participants also showed significantly fewer police contacts resulting in custody time compared to when they were off the MAP and compared to the controls. Critically, consistent changes in police and health-care-related outcomes were reported by staff and participants in the qualitative interviews. These indicators of decreases in overall hospitalization and police contacts as well as the reduction in detoxification episodes echo Thornquist et al.’s [21] findings in their evaluation of programs providing housing to severely alcohol dependent and homeless individuals as well as Podymow et al.’s [19] findings in their evaluation of the MAP in Ottawa.

It is worth stressing that the reductions in hospital admissions, admissions for detoxification, police contacts, and being taken into custody by police would have resulted in some economic savings for the local community. While not specifically measured in this pilot study, it does echo some of the same types of economic savings reported by Larimer et al. [18] in their evaluation of a Housing First program in Seattle. Further, the qualitative evidence provides an understanding of MAPs as a safer alternative than streets, police custody, or jails, and that MAPs provide critical opportunities for improving access to primary care services and addressing health issues more effectively.

### Limitations

Being a pilot study limited to a single intervention site, numbers of both MAP and control participants were relatively low. This was especially so for the handful of each group who were successively followed up for 6 months. The small sample size made it more difficult to test for significant differences between the MAP participants and the controls as well as conducting analyses by gender. There were some gaps in data for the alcohol consumption records kept by the program staff, making comparisons over the 6-month period for alcohol consumed on versus off the MAP more difficult. However, the number of different types of measures and indicators employed, and consistency between quantitative and qualitative measures gives some indication of possible benefits attributable to participation in the MAP. This study was not randomized; therefore, generalizations to other populations and to other MAPs are somewhat limited.

## Conclusions

In summary, in this small pilot study, there was evidence of substantial reductions in police contacts, police contacts leading to custody time, detoxification episodes, use of NBA, alcohol-related harms, and hospital admissions associated with MAP participation. Despite limited statistical power, some of these changes were significant, either in comparison with a similar population of control individuals or with rates of problems prior to MAP participation. The qualitative interviews indicated that perceptions of staff and experiences of participants support and provide insight into these positive outcomes. Against this overall pattern of improvement in formal indicators of alcohol-related harms is the overarching achievement of creating a safe and stable environment for a population impacted by structural vulnerabilities such as homelessness and poverty. These preliminary findings encourage the view that regulated administration of alcohol in conjunction with provision of stable housing may lead to improvements in a variety of domains not only for the MAP participants but also for the surrounding community. These issues and others such as gender differences are being explored further in a larger multi-site study across five Canadian sites.
